# Histone hyperacetylation mediates enhanced IL‐1β production in LPS/IFN‐γ‐stimulated macrophages

**DOI:** 10.1111/imm.13183

**Published:** 2020-04-07

**Authors:** Zhen Dong, Ruoshui Li, Lei Xu, Kaiyue Xin, Yamei Xu, Haiming Shi, Aijun Sun, Junbo Ge

**Affiliations:** ^1^ Department of Cardiology Zhongshan Hospital Fudan University Shanghai Institute of Cardiovascular Diseases Shanghai China; ^2^ Department of Cardiology Huashan Hospital Fudan University Shanghai China; ^3^ Institute of Biomedical Science Fudan University Shanghai China

**Keywords:** glycolysis, HDAC inhibitor, histone hyperacetylation, IL‐1β, macrophage

## Abstract

Under the condition of lipopolysaccharide (LPS)/interferon (IFN)‐γ activation, macrophage metabolism is converted from oxidative phosphorylation to glycolysis. In the present work, we analysed whether glycolysis could affect interleukin (IL)‐1β expression through altering histone acetylation levels in mouse bone marrow‐derived macrophages. Immunocytochemistry and Western blot analysis are used to characterize histone acetylation in macrophages stimulated by LPS/IFN‐γ. Real‐time polymerase chain reaction and enzyme‐linked immunosorbent assay were used to determine IL‐1β production. The metabolism of macrophages was monitored in real‐time by the Seahorse test. Our results showed that glycolytic metabolism could enhance histone acetylation and promote IL‐1β production in LPS/IFN‐γ‐activated macrophages. Moreover, increased production of IL‐1β by glycolysis was mediated through enhanced H3K9 acetylation. Importantly, it was found that a high dose of histone deacetylase inhibitor could also significantly increase the expression of IL‐1β in the absence of glycolytic metabolism. In conclusion, this study demonstrates that glycolytic metabolism could regulate IL‐1β expression by increasing histone acetylation levels in LPS/IFN‐γ‐stimulated macrophages.

## Graphical abstract

Macrophage glycolysis conversion mediates histone hyperacetylation. Histone hyperacetylation could enhance IL‐1β production. Glycolytic metabolism regulates IL‐1β expression by increasing histone acetylation levels in LPS/IFN‐γ‐stimulated macrophages.

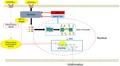

**Abbreviations**
2DG: 2‐deoxy‐d‐glucose
BMDM: bone marrow‐derived macrophage
HDAC: histone deacetylase
HIF‐1α: hypoxia‐inducible factor‐1α
IFN‐γ: interferon‐γ
IL‐1β: interleukin‐1β
LPS: lipopolysaccharide
PKM2: pyruvate kinase M2
SAHA: vorinostat
TSA: trichostatin A

## Introduction

M1 macrophages, which are transformed from activated resting state macrophages (M0) under inflammation and infection, are a type of immune cells, and play important roles in anti‐infection and promoting inflammation.^1^ In recent years, it was found that the metabolism of M1 macrophages was mainly mediated through aerobic glycolysis, characterized by significantly reduced metabolic energy supply from oxidative phosphorylation.^2^, ^3^ This kind of metabolic pattern was believed to provide energy more quickly, as well as reducing equivalent (e.g. NADH, etc.) for cells more efficiently to match the rapid response for infection and inflammation in M1 macrophages.^1^, ^4^ The metabolic pattern of macrophages and its close relationship with specific cell functions have attracted great attention in recent years.^5^, ^6^, ^7^, ^8^, ^9^

Interleukin‐1β (IL‐1β) is mainly secreted by M1 macrophages, which could regulate the expression of a variety of inflammatory factors, such as tumour necrosis factor‐α (TNF‐α), IL‐6, etc. and is in the central position in inflammatory response.^4^, ^10^ Regulation of IL‐1β expression was also a therapeutic target for many immune‐related diseases.^10^ Several studies showed that glycolytic metabolism was not only a means of rapid energy supply for M1 macrophages, but also closely associated with the regulation of IL‐1β expression.^11^, ^12^ Under the condition of glycolytic metabolism, a variety of glycolysis‐associated IL‐1β transcription factors, such as hypoxia‐inducible factor 1 (HIF‐1α) and pyruvate kinase M2 (PKM2), could facilitate the translocation into the nucleus and initiate the transcription process.^11^, ^12^ Glycolysis inhibition was linked with significantly downregulated IL‐1β expression.^11^

Histone acetylation modification is one of the epigenetic mechanisms, which could upregulate or downregulate the expression of related genes by altering chromatin structures.^13^, ^14^, ^15^, ^16^ Recent studies showed that IL‐1β expression could be regulated by histone acetylation in immune cells.^17^ It was found^18^ that IL‐1β expression was significantly increased in macrophages with ornithine decarboxylase defect due to the elevated histone H3K9 acetylation level. It is known that glycolysis metabolism is closely associated with the cell acetylation modification.^19^ In the process of glycolysis metabolism, in addition to producing a large amount of pyruvate (one of the main sources of acetyl coenzyme A), lactic acid (the main product of glycolytic metabolism) was also produced, which was a known histone deacetylase (HDAC) inhibitor.^19^ Thus, glycolytic metabolism may affect the chromatin structure, especially the histone acetylation level in M1 macrophages. In addition, glycolysis also affects IL‐1β‐related transcription factors, but it remains largely unclear whether glycolysis could also directly affect IL‐1β transcription by modifying histone structures (especially the histone acetylation level).

In this study, we showed that glycolysis could regulate the acetylation level of H3K9 in macrophages, and then affect the IL‐1β expression. Our results thus revealed that H3K9 hyperacetylation was an important initial mediator for inducing IL‐1β upregulation, and this effect is independent on glycolysis and hyperacetylation could promote IL‐1β transcriptional levels even in the case of inhibited glycolysis conversion.

## Materials and methods

#### Materials/reagents

All reagents used for cell culture were from Gibco. Reagents for RNA extraction, cDNA synthesis and real‐time polymerase chain reaction (RT‐PCR) were purchased from TAKARA. The following reagents were obtained from Novoprotein: murine recombinant M‐CSF, murine recombinant interferon (IFN)‐γ. Lipopolysaccharide (LPS) from *Escherichia coli* O111:E4 was obtained from Sigma‐Aldrich. The following reagents were obtained from MedChemExpress: SAHA (vorinostat), TSA (trichostatin A) and 2DG (2‐deoxy‐d‐glucose).

#### Isolation of bone marrow‐derived macrophages

Bone marrow‐derived macrophages (BMDMs) were isolated and differentiated as previously described.^14^, ^18^, ^20^ Upon completion of differentiation, cells（1·5–2 × 10^6^ cells/well） were placed in Dulbecco’s modified Eagle’s medium (DMEM)/F12, supplemented with 10% [50 ml fetal bovine serum (FBS)/500 ml DMEM/F12] FBS, 2 mm l‐glutamine and 15 mm HEPES for the next experiments.

#### Cell culture and treatments

To study the effect of 2DG on LPS/IFN‐γ stimulated BMDMs, cells were incubated with vehicle or increasing concentrations of 2DG 0·1, 1 or 5 mm combined with LPS (100 ng/ml)/IFN‐γ (10 ng/ml) for 12 hr.^21^ For time course experiments, stimulated BMDMs were treated with vehicle or 2DG 5 mm, and samples collected at 12 hr and 24 hr. For HDAC inhibition experiments, stimulated macrophages were treated with SAHA (0·1 or 0·5μm) or TSA (0·1 μm) with or without 2DG (5 mm) for 12 hr.

#### RT‐quantitative (q)PCR

For total RNA isolation, 12‐ or 24‐hr‐treated BMDMs were extracted using TRIzol reagent, and reverse transcription was performed using the PrimeScript™ RT reagent Kit according to the manufacturers' instructions (Takara Biomedical Technology‚ Dalian‚ China). RT‐qPCR was performed in a CFX96 Touch™ Real‐Time PCR Detection System (Bio‐Rad Laboratories, Hercules, CA, USA) with SYBR^®^ Premix Ex TaqTM II (Takara Biomedical Technology) using the following primers:^22^ IL‐1β: forward 5′‐TTCAGGCAGGCAGTATCACTCATTG‐3′, reverse 5′‐ACACCAGCAGGTTATCATCATCATCC‐3′; ND1: forward 5′‐CTCAACCTAGCAGAAACAAACC‐3′, reverse 5′‐GGCCGGCTGCGTATTCTAC‐3′; ND6: forward 5′‐TTGGGAGATTGGTTGATGTAT‐3′, reverse 5′‐TGCCGCTACCCCAATCC‐3′; Cox1: forward 5′‐TCAGTATCGTATGCTTCAACAAATTTAGA‐3′, reverse 5′‐TGGTTCCTCGAATGTGTGATATG‐3′; Cox2: forward 5′‐GAGCAGTCCCCTCCCTAGGA‐3′, reverse 5′‐GTCGGTTTGATGTTACTGTTGCTT‐3′; cytochrome *b*: forward 5′‐AAAGCCACCTTGACCCGATT‐3′, reverse 5′‐GATTCGTAGGGCCGCGATA‐3′; ATPase6: forward 5′‐TCGTTGTAGCCATCATTATATTTCCT‐3′, reverse 5′‐GAAAGAATGGAGACGGTTGTTGA‐3′; 16SRNA: forward 5′‐TGCCTGCCCAGTGACTAAAGT‐3′, reverse 5′‐AACAAGTGATTATGCTACCTTTGCA‐3′; tRNA leucine 1: forward 5′‐GGTGGCAGACGGAGGAAA‐3′, reverse 5′‐TATTAGGGAGAGGATTTGAACCT‐3′; HDAC4: forward 5′‐CACTGCATTTCCAGCGATCC‐3′, reverse 5′‐AAGACGGGGTGGTTGTAGGA‐3′; HDAC2: forward 5′‐GGAGGAGGCTACACAATCCG‐3′, reverse 5′‐TCTGGAGTGTTCTGGTTTGTCA‐3′; HDAC1: forward 5′‐AGTCTGTTACTACTACGACGGG‐3′, reverse 5′‐TGAGCAGCAAATTGTGAGTCAT‐3′; HDAC3: forward 5′‐GCCAAGACCGTGGCGTATT‐3′, reverse 5′‐GTCCAGCTCCATAGTGGAAGT‐3′; HDAC6: forward 5′‐TCCACCGGCCAAGATTCTTC‐3′, reverse 5′‐CAGCACACTTCTTTCCACCAC‐3′; HDAC5: forward 5′‐TGCAGCACGTTTTGCTCCT‐3′, reverse 5′‐GACAGCTCCCCAGTTTTGGT‐3′; HDAC7: forward 5′‐GAACTCTTGAGCCCTTGGACA‐3′, reverse 5′‐GGTGTGCTGCTACTACTGGG‐3′; HDAC9: forward 5′‐GCGGTCCAGGTTAAAACAGAA‐3′, reverse 5′‐GCCACCTCAAACACTCGCTT‐3′. Levels of mRNA for specific genes were shown as relative gene expression normalized to β‐actin.

#### Metabolism assays

Real‐time measurements of oxygen consumption rate (OCR) and extracellular acidification rate (ECAR) were performed as previously described^23^, ^24^ using a Seahorse XF96 Extracellular Flux Analyzer (Seahorse Bioscience, billerica, MA, USA). Differentiated BMDMs (5 × 10^5^ cell/ml) were seeded as a monolayer in a 96‐well microplate containing XF Assay Modified Culture Media. Metabolic substances were injected during real‐time measurements of OCR and ECAR according to the manufacturers’ instructions. Maximal respiration: the maximum rate measurement after addition of FCCP – non‐mitochondrial respiration (minimum rate measurement after Rot/AntA); Glycolysis: ECAR values before the addition of oligomycin – ECAR values before the addition of glucose; Glycolytic capacity: ECAR values following addition of oligomycin – ECAR values before the addition of glucose. Levels of OCR and ECAR were shown as relative values normalized by protein concentrations.^23^

#### Immunocytochemistry

Cells were fixed and permeabilized by incubating in ice‐cold methanol at −20° for 15 min, and then washed in phosphate‐buffered saline (PBS) for 5 min three times. Samples were blocked in PBS containing 5% bovine serum albumin (BSA; Sigma‐Aldrich,Darmstadt, Germany) for 30 min at room temperature, and incubated with the primary antibody diluted in PBS containing 3% BSA at 4° for 12 hr. Samples were washed three times with PBS, incubated with the secondary antibody diluted in PBS at room temperature for 1·5 hr, and washed with PBS three times. The nucleus was labelled by DAPI (4′,6‐diamidino‐2‐phenylindole; Sigma‐Aldrich). Primary antibody: Histone H3 antibody (1:100, #4499) and Acetyl‐Histone H3 (Lys9) (1:100, #9649) were purchased from Cell Signaling Technology; Anti‐Monocyte + Macrophage antibody (MOMA‐2) (1:100, ab33451) was purchased from Abcam. Secondary antibody were purchased from Invitrogen.

#### Western blot analysis

Cells were lysed in RIPA supplemented with protease and phosphatase inhibitors (Thermo Fisher Scientific, Waltham, MA, USA), and total protein content was analysed by the BCA method (Beyotime Biotechnology, Shanghai, China). Lysate proteins were separated by 10%−12% Criterion TGX Stain‐Free Gel (Bio‐Rad), and were transferred to PVDF membranes with the Trans‐Blot Turbo Transfer system (Bio‐Rad). Membranes were blocked for 1 hr with 5% BSA diluted in TBS containing 0·1% Tween 20, and then incubated for 12 hr at 4° with the primary antibody. Membranes were washed and incubated for 2 hr with horseradish peroxidase (HRP)‐coupled goat polyclonal anti‐rabbit IgG or HRP‐coupled goat polyclonal anti‐mouse IgG (Biotechwell Biotechnology, Shanghai, China). Immunoblotting was conducted using SuperSignal West Femto (Thermo Fisher Scientific). Primary antibody: Histone H3 antibody (1:1000, #4499), Acetyl‐Histone H3 (Lys9) (1:1000, #9649) and COX IV antibody (1:1000, #11967) were purchased from Cell Signaling Technology; HIF‐1a antibody (1:800, NB100‐105) was purchased from Novus Biologicals; Glut‐1 antibody (1:5000, ab115730) and VDAC antibody (1:2000, ab14734) were purchased from Abcam.

#### Sample preparation and GC‐MS for metabolic flux analysis

Upon completion of differentiation, BMDMs were pre‐incubated with vehicle, 2DG 5 mm or SAHA 0·5 μm for 4 hr, and then the media was replaced with glucose‐free DMEM (Biological Industries, Shanghai, China) supplemented with 17.5 mm [U‐^13^C_6_]‐glucose (Sigma‐Aldrich) and 10% FBS combined with LPS (100 ng/ml)/IFN‐γ (10 ng/ml) for 12 hr. Stimulated macrophages were still treated with 2DG (5 mm) or SAHA (0·5 μm) for 12 hr. After 12 hr of LPS/IFN‐γ stimulation, cell samples collected in 600 μl of 50% methanol solution were supplemented with 1200 μl methanol before being processed by five cycles of 2 min ultra‐sonication and 2 min interval in ice‐water bath. After centrifugation at 16 000 ***g*** and 4° for 15 min, 250 μl of supernatant and 10 μl internal standards (50 μg/ml l‐norvaline) were mixed and evaporated to dryness under nitrogen stream. The dry residue was reconstituted in 30 μl of 20 mg/ml methoxyamine hydrochloride in pyridine, and the resulting mixture was incubated at 37° for 90 min. Then, 30 μl of MTBSTFA (with 1% TBDMS) was added into the mixture and derivatized at 55° for 60 min prior to GC‐MS metabolomics analysis. Instrumental analysis was performed on an Agilent 7890A gas chromatography system coupled to an Agilent 5975C inert MSD system (Agilent Technologies, Santa Clara, CA, USA). An OPTIMA^®^ 5 MS Accent fused‐silica capillary column (30 m × 0·25 mm × 0·25 μm; MACHEREY‐NAGEL, Düren, GERMAN) was utilized to separate the derivatives. Helium (> 99·999%) was used as a carrier gas at a constant flow rate of 1 ml/min through the column. Injection volume was 1 μl, and the solvent delay time was 5·9 min. The initial oven temperature was held at 100° for 2 min, ramped to 170° at a rate of 10°/min, to 260° at a rate of 15°/min, to 320° at a rate of 30°/min, and finally held for 5 min. The temperatures of injector, transfer line and electron impact ion source were set to 250°, 260° and 230°, respectively. The electron energy was 70 eV, and data were collected in a full‐scan mode (m/z 50–600). Steady‐state metabolic flux was calculated by ^13^C mass isotopologue distributions (MIDs) for acetate, citrate, pyruvate and lactate with home‐made package in R language, which applies an elementary metabolite unit framework to efficiently simulate MIDs and deducts natural isotope abundance.

#### Enzyme‐linked immunosorbent assay (ELISA) assays

Culture supernatants were collected after treatment and stored at −80°. IL‐1β was measured using Mouse IL‐1β ELISA Kit (Company ABclonal) according to the manufacturer's protocol.

#### Statistical analysis

Multiple group comparisons were performed by one‐way anova followed by Tukey–Kramer *post hoc* analysis. The data are presented as means ± SD. Differences were considered statistically significant with *P* < 0·05.

## Results

### LPS/IFN‐γ stimulated the glycolytic metabolism conversion in activated macrophages and histone H3K9 hyperacetylation

**Figure 1:**
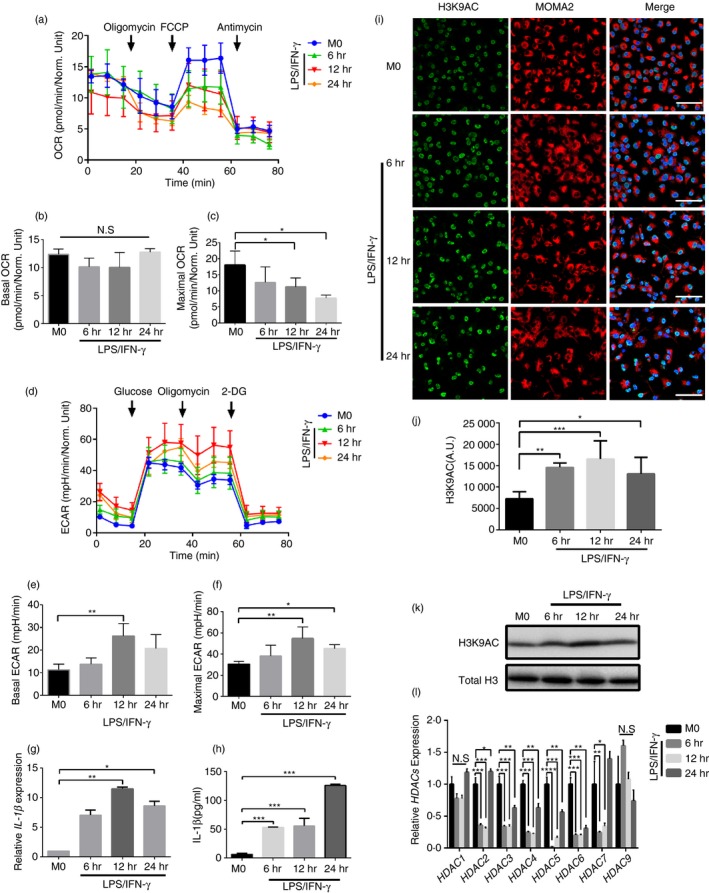
Histone hyperacetylation and metabolic reprogramming during differentiation of macrophages activated by lipopolysaccharide (LPS)/interferon (IFN)‐γ. (a) Summarized curves of oxygen consumption rate (OCR) tracings from Seahorse experiments for bone marrow‐derived macrophages (BMDMs) at the indicated differentiation stages activated by LPS/IFN‐γ. (b,c) Quantification of basal(F) and maximal(G) OCR in BMDMs at the indicated differentiation stages activated by LPS/IFN‐γ. *n* = 3 independent measurements. *P < 0·05. Data are presented as mean ± SD. (d) Summarized curves of extracellular acidification rate (ECAR) tracings from Seahorse experiments for BMDMs at the indicated differentiation stages activated by LPS/IFN‐γ. (e,f) Quantification of basal (J) and maximal (K) ECAR in BMDMs at the indicated differentiation stages activated by LPS/IFN‐γ. *n* = 3 independent measurements. *P < 0·05. Data are presented as mean ± SD. (g) mRNA levels of *IL‐1β* were assessed by real‐time polymerase chain reaction (RT‐PCR) in BMDMs activated by LPS/IFN‐γ. *P < 0·05, **P < 0·01, ***P < 0·001, ****P < 0·0001. All samples were analysed as fold change against M0 inactivated control samples. *n* = 3 independent measurements. Data are presented as mean ± SD. (h) Interleukin (IL)‐1β was quantified by enzyme‐linked immunosorbent assay (ELISA) in BMDMs culture supernatants collected at the indicated differentiation stages activated by LPS/IFN‐γ. ***P < 0·001. *n* = 3 independent measurements. Data are presented as mean ± SD. (i) Representative immunofluorescence images of histone acetylation at histone 3, lysine 9 (H3K9) during differentiation of BMDMs activated by LPS/IFN‐γ. Green, AC‐H3K9; red, MOMA2 and blue, DAPI. A.U. indicates arbitrary units. Scale bar: 50 μm. (j) Quantification of AC‐H3K9 immunostaining in BMDMs as in (a) at the indicated differentiation stages activated by LPS/IFN‐γ. *P < 0·05, **P < 0·01, ***P < 0·001. *n* = 3 independent measurements. Data are presented as mean ± SD. (k) Representative Western blotting (WB) of AC‐H3K9 in BMDMs at the indicated differentiation stages activated by LPS/IFN‐γ. *n* = 3 independent measurements. (l) mRNA levels of *HDACs 1‐7,9* were assessed by RT‐PCR in BMDMs activated by LPS/IFN‐γ. *P < 0·05, **P < 0·01, ***P < 0·001. All samples were analysed as fold change against M0 inactivated control samples. *n* = 3 independent measurements. Data are presented as mean ± SD.

Consistent with previous reports, under the stimulation of LPS‐induced IFN‐gamma (LPS/IFN‐γ), the metabolic status of resting state macrophages gradually converted from oxidative phosphorylation to glycolytic metabolism (Figs 1a–f and S1a–c).^5^, ^11^ At the same time, IL‐1β expression increased at both mRNA and protein levels (Fig. 1g,h) as glycolysis level increased. Besides, it was also observed that acetylation of total proteins and H3K9 both increased with the increase of glycolysis by using methods of immunofluorescence and Western blotting (WB; Figs 1i–k and S1d). The same changes of glycolysis and acetylation of H3K9 were observed in macrophages separately stimulated by LPS and IFN‐γ (Fig. S2a–e). As total and histone acetylation were negatively regulated by HDACs family, RT‐PCR was used to detect the expression changes of class I and class II HDACs. Corresponding to the increase of histone acetylation levels, compared with resting macrophages (M0), the expression of class I and class II HDACs significantly decreased under LPS/IFN‐γ stimulation (except HDAC1 and 9; Fig. 1l).

**Figure 2:**
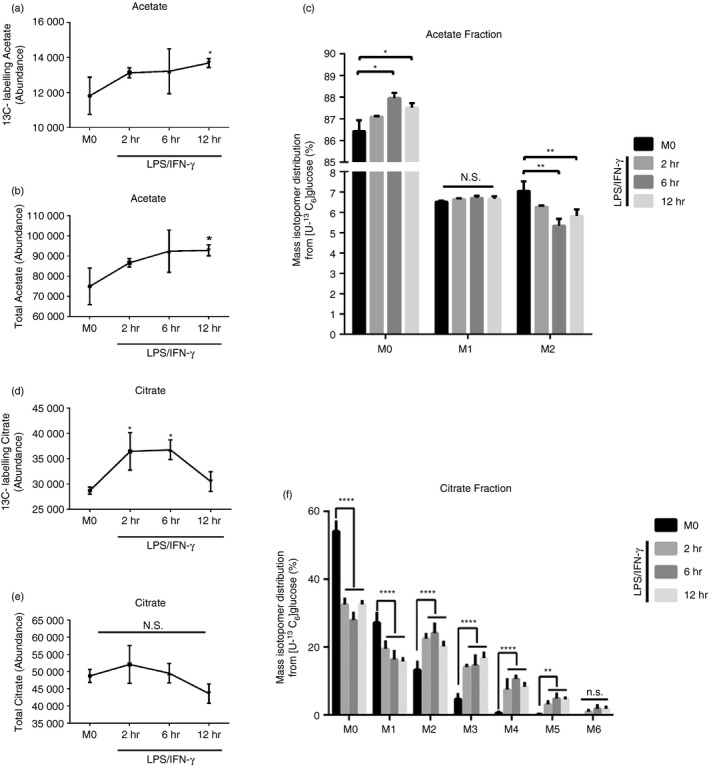
Changes of cellular acetate and citrate content during glycolytic transformation in lipopolysaccharide (LPS)/interferon (IFN)‐stimulated macrophages. (a,b) Cellular ^13^C‐labelling and total acetate content examined by GC‐MS in LPS/IFN‐stimulated macrophages. (c) Mass isotopologue analysis of acetate in LPS/IFN‐stimulated macrophages cultured with [U‐13C6]‐glucose. M (0‐2): the number of labelled carbons; *P < 0·05, **P < 0·01; *n* = 3 cultures from a representative experiment. (d,e) Cellular ^13^C‐labelling and total citrate content examined by GC‐MS in LPS/IFN‐stimulated macrophages. (f) Mass isotopologue analysis of citrate in LPS/IFN‐stimulated macrophages cultured with [U‐13C6]‐glucose. M (0–6): the number of labelled carbons; *P < 0·05, **P < 0·01, ****P < 0·0001; *n* = 3 cultures from a representative experiment; Data are presented as mean ± SD.

In addition to the regulatory role of HDACs family, histone acetylation level can also be affected by the availability of acetyl‐CoA donors (acetate and citrate).^25^, ^26^ Therefore, we examined the alterations of acetate and citrate, as well as important glycolysis metabolites (such as pyruvate and lactate) in activated macrophages by mass isotopologue analysis. It was revealed that both labelled/total acetate and labelled citrate increased with the prolongation of LPS/INF‐γ stimulation (Fig. 2a,b,d–e); the pyruvate and lactate contents also showed the same increasing alterations (Fig. S3a,b,d,e). However, to our surprise, we found that the content of unlabelled acetate was much higher than that of labelled (Fig. 2c), which was inconsistent with the changes in citrate, pyruvate and lactate (Figs 2f and S3c,f). According to these observations, we demonstrated that glycolytic metabolic conversion of macrophages is closely related to the acetylation of total and histone proteins through regulating HDACs and acetyl‐CoA donors.

### Glycolysis was an initial mediator for IL‐1β expression and H3K9 hyperacetylation in LPS/IFN‐γ‐stimulated macrophages

**Figure 3:**
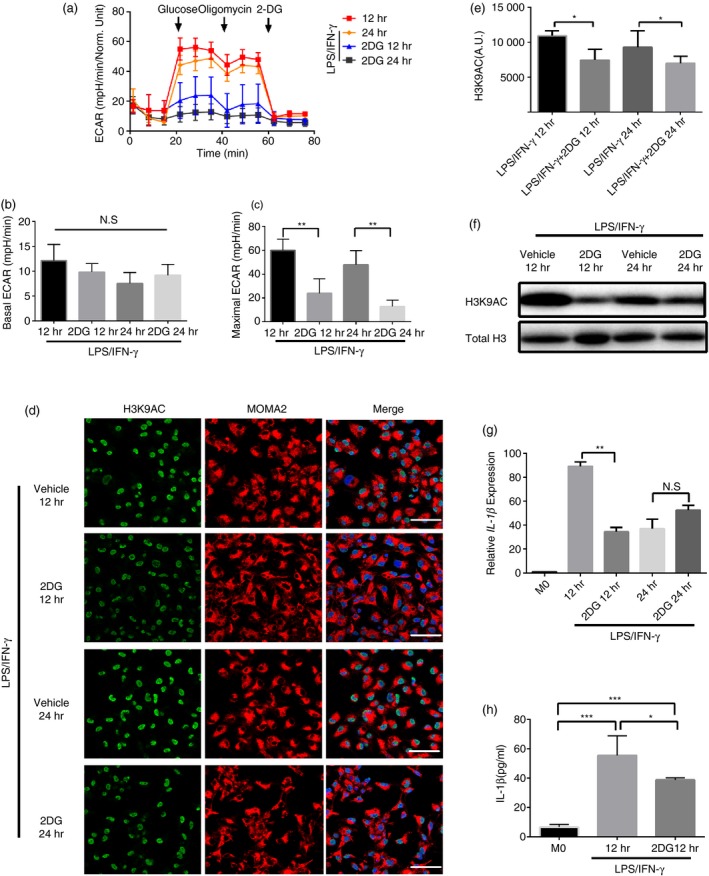
Glycolysis is necessary for lipopolysaccharide (LPS)/interferon (IFN)‐γ‐induced interleukin (IL)‐1β expression and H3K9 hyperacetylation. (a) Summarized curves of extracellular acidification rate (ECAR) tracings from Seahorse experiments for bone marrow‐derived macrophages (BMDMs) at the indicated differentiation time points activated by LPS/IFN‐γ with vehicle [phosphate‐buffered saline (PBS)] or 5 mm 2‐deoxy‐d‐glucose (2DG). (b,c) Quantification of basal (E) and maximal (F) ECAR in BMDMs at the indicated differentiation time points activated by LPS/IFN‐γ with vehicle (PBS) or 5 mm 2DG. *n* = 3 independent measurements. **P < 0·01. Data are presented as mean ± SD. (d) Representative immunofluorescence images of AC‐H3K9 during differentiation of BMDMs activated by LPS/IFN‐γ 12 hr and 24 hr with vehicle (PBS) or 5 mm 2DG. Green, AC‐H3K9; red, MOMA2 and blue, DAPI. A.U. indicates arbitrary units. Scale bar: 50 μm. (e) Quantification of AC‐H3K9 immunostaining in BMDMs as in (a) at the indicated differentiation time points activated by LPS/IFN‐γ with vehicle (PBS) or 5 mm 2DG. *P < 0·05. *n* = 3 independent measurements. Data are presented as mean ± SD. (f) Representative Western blot (WB) of AC‐H3K9 in BMDMs at the indicated differentiation time points activated by LPS/IFN‐γ with vehicle (PBS) or 5 mm 2DG. *n* = 3 independent measurements. (g) mRNA levels of *IL‐1β* were assessed by real‐time polymerase chain reaction (RT‐PCR) in BMDMs activated by LPS/IFN‐γ with vehicle (PBS) or 5 mm 2DG. *n* = 3 independent measurements. **P < 0·01. All samples were analysed as fold change against M0 inactivated control samples. Data are presented as mean ± SD. (h) IL‐1β was quantified by enzyme‐linked immunosorbent assay (ELISA) in BMDMs culture supernatants collected at 12 hr activated by LPS/IFN‐γ with vehicle (PBS) or 5 mm 2DG. *P < 0·05, ***P < 0·001. *n* = 3 independent measurements. Data are presented as mean ± SD.

**Figure 4:**
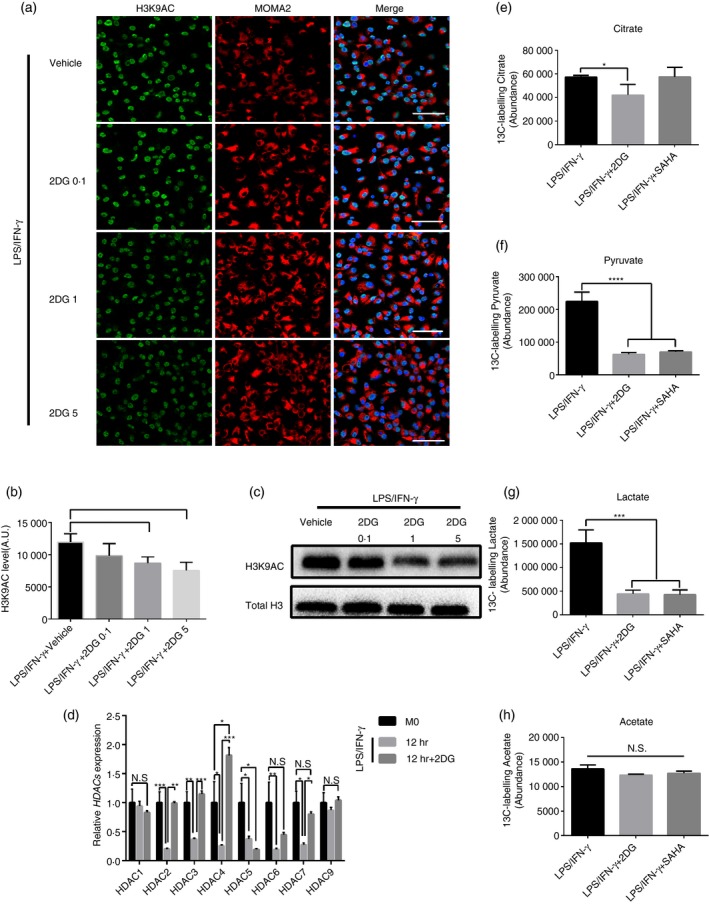
Dose‐dependent effects of 2‐deoxy‐d‐glucose (2DG) on acetylation level of H3K9 and interleukin (IL)‐1β expression. (a) Representative immunofluorescence images of AC‐H3K9 during differentiation of bone marrow‐derived macrophages (BMDMs) induced by lipopolysaccharide (LPS)/interferon (IFN)‐γ 12 hr with or without 2DG (0·1, 1, 5 mm). Green, AC‐H3K9; red, MOMA2 and blue, DAPI. A.U. indicates arbitrary units. Scale bar: 50 μm. (b) Quantification of AC‐H3K9 immunostaining in BMDMs as in (a) induced by LPS/IFN‐γ 12 hr with or without 2DG (0·1, 1, 5 mm). *P < 0·05, ***P < 0·001. *n* = 3 independent measurements. Data are presented as mean ± SD. (c) Representative Western blot (WB) of AC‐H3K9 in BMDMs at induced by LPS/IFN‐γ 12 hr with or without 2DG (0·1, 1, 5 mm). *n* = 3 independent measurements. (d) mRNA levels of histone deacetylases (HDACs) 1–7, 9 were assessed by real‐time polymerase chain reaction (RT‐PCR) in BMDMs activated by LPS/IFN‐γ with vehicle [phosphate‐buffered saline (PBS)] or 5 mm 2DG. *P < 0·05, **P < 0·01, ***P < 0·001. All samples were analysed as fold change against M0 inactivated control samples. *n* = 3 independent measurements. Data are presented as mean ± SD. (e–h) Quantification of cellular ^13^C‐labelling citrate, pyruvate, lactate and acetate examined by GC‐MS in macrophages stimulated by LPS/IFN‐γ 12 hr combined with 2DG (5 mm) or vorinostat (SAHA; 0·5 μm). *P < 0·05, ***P < 0·001, ****P < 0·0001. *n* = 3 cultures from a representative experiment. Data are presented as mean ± SD.

Previous studies revealed that metabolism could affect transcription of inflammation‐related genes by influencing chromatin structures (including histone modification).^25^ More specifically, the study by Hardbower *et al.* ^18^ showed that H3K9 hyperacetylation promoted the transcription and expression of IL‐1β. Based on the evidence in Figs 1 and 2, it was proposed that the enhancement of glycolysis mediated the increase of H3K9 acetylation level. To verify this hypothesis, classic glycolysis 2DG^27^ was used to observe the effects of glycolysis on the changes of H3K9 acetylation and IL‐1β expression. In accordance with expectations, 2DG could significantly inhibit LPS/IFN‐γ‐mediated glycolysis level in macrophages (Fig. 3a–c), and the acetylation level of H3K9 was decreased (Fig. 3d–f). Similarly, IL‐1β expression was also decreased (Fig. 3g,h). Furthermore, it was revealed that 2DG reduced the acetylation level of H3K9 and IL‐1β expression in a dose‐dependent manner (Fig. 4a–c). More interestingly, 2DG treatment restored the expression of HDACs (except HDAC5 and 6) inhibited by increased glycolysis, suggesting that glycolysis could regulate the expression of HDACs in macrophages activated by LPS/IFN‐γ (Fig. 4d). Moreover, 2DG treatment significantly reduced C13‐labelled citrate, pyruvate and lactate levels in activated macrophages, although the production of labelled acetate was not significantly altered by the intervention (Fig. 4e–h). The study by Wang *et al.* ^12^ showed that *Sirt5* knockout (KO) macrophages exhibited enhanced glycolytic metabolism under the stimulation of LPS. Therefore, we also examined the acetylation level of H3K9 and the expression of IL‐1β in *Sirt5* KO macrophages stimulated by LPS/IFN‐γ. Interestingly, acetylation of H3K9 increased with the increase of glycolysis (Fig. S4a–c). Similarly, IL‐1β expression increased as well in *Sirt5* KO macrophages stimulated by LPS/IFN‐γ (Fig. S4d). According to these observations, we demonstrated that H3K9 acetylation and IL‐1β expression were regulated by glycolytic cycle.

### The inhibitory effect of 2DG on IL‐1β expression in M1 macrophages depended on the decrease of histone acetylation level

**Figure 5:**
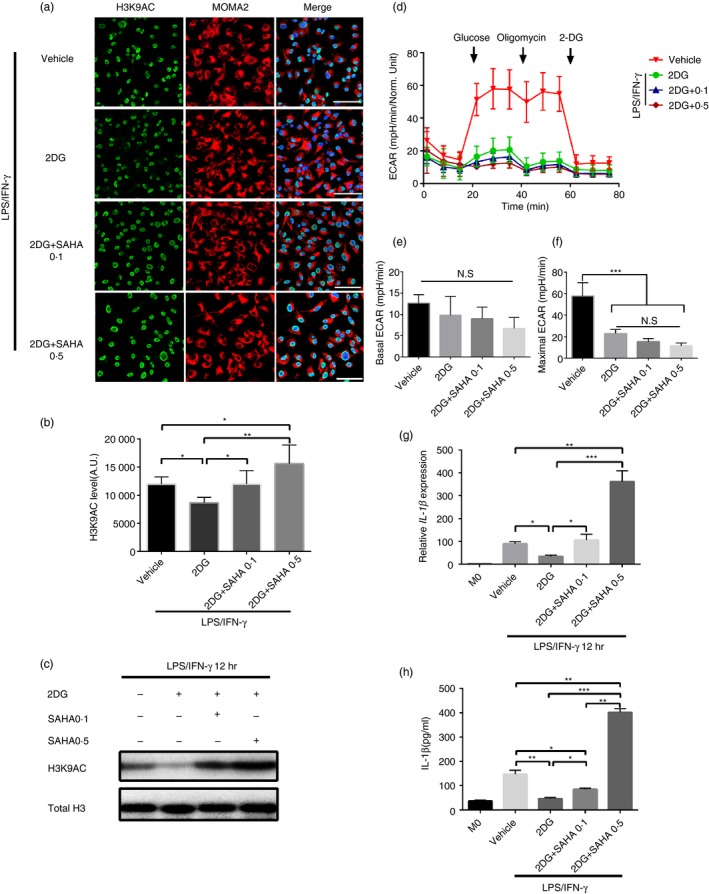
The inhibitory effect of 2‐deoxy‐d‐glucose (2DG) on interleukin (IL)‐1β expression depends on the acetylation level of H3K9. (a) Representative immunofluorescence images of AC‐H3K9 during differentiation of bone marrow‐derived macrophages (BMDMs) activated by lipopolysaccharide (LPS)/interferon (IFN)‐γ 12 hr with vehicle, 5 mm 2DG and 5 mm 2DG + vorinostat (SAHA; 0·1, 0·5 μm). Green, AC‐H3K9; red, MOMA2 and blue, DAPI. A.U. indicates arbitrary units. Scale bar: 50 μm. (b) Quantification of AC‐H3K9 immunostaining in BMDMs as in (a) at induced by LPS/IFN‐γ 12 hr with vehicle, 5 mm 2DG and 5 mm 2DG + SAHA (0·1, 0·5 μm). *P < 0·05, **P < 0·01. *n* = 3 independent measurements. Data are presented as mean ± SD. (c) Representative Western blot (WB) of AC‐H3K9 in BMDMs induced by LPS/IFN‐γ 12 hr with vehicle, 5 mm 2DG and 5 mm 2DG + SAHA (0·1, 0·5 μm). *n* = 3 independent measurements. (d) Summarized curves of extracellular acidification rate (ECAR) tracings from Seahorse experiments for BMDMs activated by LPS/IFN‐γ 12 hr with vehicle, 5 mm 2DG and 5 mm 2DG + SAHA (0·1, 0·5 μm). (e,f) Quantification of basal (E) and maximal (F) ECAR in BMDMs activated by LPS/IFN‐γ 12 hr with vehicle, 5 mm 2DG and 5 mm 2DG + SAHA (0·1, 0·5 μm). *n* = 3 independent measurements. ***P < 0·001. Data are presented as mean ± SD. (g) mRNA levels of *IL‐1β* were assessed by real‐time polymerase chain reaction (RT‐PCR) in BMDMs activated by LPS/IFN‐γ 12 hr with vehicle, 5 mm 2DG and 5 mm 2DG + SAHA (0·1, 0·5 μm). *n* = 3 independent measurements. *P < 0·05, **P < 0·01, ***P < 0·001. All samples were analysed as fold change against M0 inactivated control samples. Data are presented as mean ± SD. (h) IL‐1β was quantified by enzyme‐linked immunosorbent assay (ELISA) in BMDMs culture supernatants collected at 12 hr activated by LPS/IFN‐γ with vehicle, 5 mm 2DG and 5 mm 2DG + SAHA (0·1,0·5 μm). *P < 0·05, **P < 0·01, ***P < 0·001. *n* = 3 independent measurements. Data are presented as mean ± SD.

Our analysis indicated that IL‐1β expression and H3K9 acetylation were simultaneously inhibited when glycolysis was inhibited. It was revealed that glycolysis inhibiting IL‐1β expression depended on the decrease of H3K9 acetylation level. Therefore, it needed to be verified whether it was possible to counteract the inhibitory effect of 2DG on IL‐1β by artificially elevating H3K9 acetylation level. To this end, 2DG and SAHA (a HDAC inhibitor used in several reports to elevate H3K9 acetylation level) were used in combination, and the changes of H3K9 acetylation and IL‐1β expression were observed. As expected, SAHA intervention could reverse the inhibitory effect of 2DG on H3K9 acetylation level (Fig. 5a–c). However, there was no significant effect on glycolysis itself, which could eliminate the possibility that SAHA might affect H3K9 acetylation level by modifying glycolysis (Fig. 5d–f). Interestingly, the inhibitory effect of 2DG on IL‐1β was gradually reversed both at mRNA and protein levels along with the increase of SAHA concentration, even though the glycolytic metabolism of macrophages was still significantly inhibited (Fig. 5g,h). In conclusion, our experiments revealed that the inhibitory effect of 2DG on IL‐1β depended on the inhibition of H3K9 acetylation level, which was achieved by inhibiting glycolysis. This also indicated that histone H3K9 hyperacetylation was indispensable for IL‐1β expression.

### Histone hyperacetylation promoted IL‐1β expression in M1 macrophages

**Figure 6:**
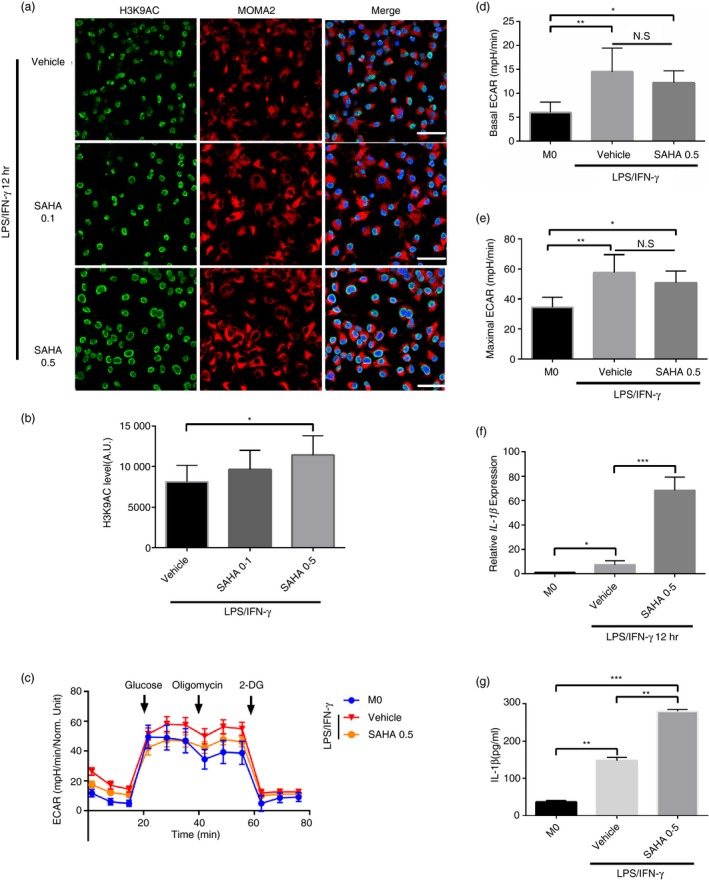
Histone hyperacetylation enhanced the expression of interleukin (IL)‐1β expression. (a) Representative immunofluorescence images of AC‐H3K9 during differentiation of bone marrow‐derived macrophages (BMDMs) activated by lipopolysaccharide (LPS)/interferon (IFN)‐γ 12 hr with vehicle or vorinostat (SAHA) 0·5 μm. Green, AC‐H3K9; red, MOMA2 and blue, DAPI. A.U. indicates arbitrary units. Scale bar: 50 μm. (b) Quantification of AC‐H3K9 immunostaining in BMDMs as in (a) induced by LPS/IFN‐γ 12 hr with vehicle or SAHA 0·5 μM. *P < 0·05. *n* = 3 independent measurements. Data are presented as mean ± SD. (c) Summarized curves of extracellular acidification rate (ECAR) tracings from Seahorse experiments for BMDMs activated by LPS/IFN‐γ 12 hr with vehicle or SAHA 0·5 μm. (d,e) Quantification of basal (E) and maximal (F) ECAR in BMDMs activated by LPS/IFN‐γ 12 hr with vehicle or SAHA 0·5 μm. *n* = 3 biological replicates. *P < 0·05, **P < 0·01. *n* = 3 independent measurements. Data are presented as mean ± SD. (f) mRNA levels of *IL‐1β* were assessed by real‐time polymerase chain reaction (RT‐PCR) in BMDMs activated by LPS/IFN‐γ 12 hr with vehicle or SAHA 0·5 μm. *n* = 3 independent measurements. *P < 0·05, ***P < 0·001. All samples were analysed as fold change against M0 inactivated control samples. Data are presented as mean ± SD. (g) IL‐1β was quantified by enzyme‐linked immunosorbent assay (ELISA) in BMDMs culture supernatants collected at 12 hr activated by LPS/IFN‐γ with vehicle or SAHA 0·5 μm. **P < 0·01, ***P < 0·001. *n* = 3 independent measurements. Data are presented as mean ± SD.

Based on the above‐mentioned observations, whether IL‐1β expression could be further promoted by elevating H3K9 acetylation level was investigated as well. Therefore, during the stimulation of macrophages with LPS/IFN‐γ, SAHA intervention was applied. By using immunofluorescence and WB, it was revealed that H3K9 acetylation level in macrophages significantly increased after SAHA stimulation (Fig. 6a,b). The increase of glycolysis in macrophages caused by LPS/IFN‐γ stimulation was not significantly affected, which excluded the possibility that SAHA might affect H3K9 acetylation level by changing glycol‐metabolism (Fig. 6c–e). Moreover, the IL‐1β expression significantly increased at both mRNA and protein levels after SAHA intervention (Fig. 6f,g). The same changes of IL‐1β expression were also observed in macrophages separately stimulated by LPS and IFN‐γ combined with or without SAHA treatment (Fig. S4e,f). In addition to IL‐1β, the expressions of TNF‐α, IL‐6 and NOS2 were also investigated under LPS/IFN‐γ stimulation (Fig. S4g–i).

### Histone hyperacetylation promoted IL‐1β expression in the absence of glycolysis metabolic conversion in M1 macrophages

**Figure 7:**
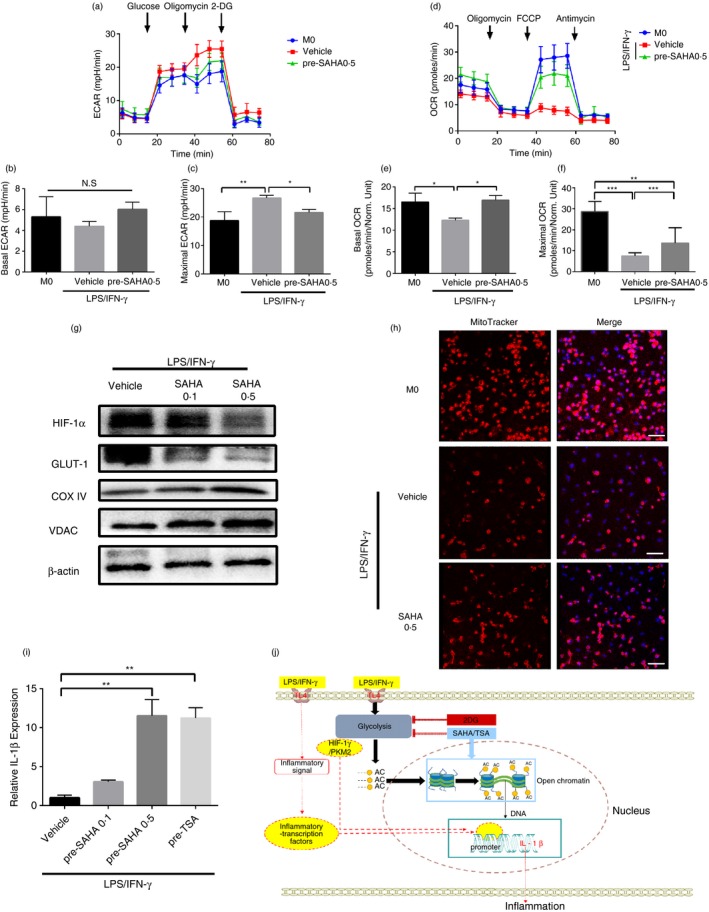
Histone hyperacetylation promoted interleukin (IL)‐1β expression in the absence of glycolysis metabolic conversion in M1 macrophages. (a) Summarized curves of extracellular acidification rate (ECAR) tracings from Seahorse experiments for bone marrow‐derived macrophages (BMDMs) 24 hr activated by lipopolysaccharide (LPS)/interferon (IFN)‐γ pretreated with vehicle or vorinostat (SAHA) 0·5 μm 6 hr. (b,c) Quantification of basal (E) and maximal (F) ECAR in BMDMs 24 hr activated by LPS/IFN‐γ pretreated with vehicle or SAHA 0·5 μm 12 hr. *n* = 3 independent measurements. *P < 0·05, **P < 0·01. Data are presented as mean ± SD. (d) Summarized curves of oxygen consumption rate (OCR) tracings from Seahorse experiments for BMDMs 24 hr activated by LPS/IFN‐γ pretreated with vehicle or SAHA 0·5 μm 12 hr. (e,f) Quantification of basal (I) and maximal (J) OCR in BMDMs 24 hr activated by LPS/IFN‐γ pretreated with vehicle or SAHA 0·5 μm 12 hr. *n* = 3 independent measurements. *P < 0·05, **P < 0·01, ***P < 0·001. Data are presented as mean ± SD. (g) Representative Western blot (WB) of hypoxia‐inducible factor‐1α (HIF‐1α), GLUT‐1, COXIV and VDAC levels in BMDMs 24 hr activated by LPS/IFN‐γ pretreated with vehicle or SAHA 0·5 μm 12 hr. *n* = 3 independent measurements. (h) Representative MitoTracker stains during differentiation of BMDMs activated by LPS/IFN‐γ pretreated with vehicle or SAHA 0·5 μm 12 hr. Red, mitochondrial; blue, DAPI. Scale bar: 50 μm. *n* = 3 independent measurements. (i) mRNA levels of *IL‐1β* were assessed by real‐time polymerase chain reaction (RT‐PCR) in BMDMs activated by LPS/IFN‐γ pretreated with vehicle, SAHA (0·1, 0·5 μm) and trichostatin A (TSA; 0·1 μm) 12 hr. *n* = 3 independent measurements. **P < 0·01. All samples were analysed as fold change against M0 inactivated control samples. Data are presented as mean ± SD. (j) Schematic description of histone hyperacetylation promoting IL‐1β expression in the in M1 macrophages activated by LPS/IFN‐γ. After LPS/IFN‐γ stimulation, macrophages undergo metabolic conversion. With the increase of glycolysis level, histone deacetylases (HDACs) were significantly inhibited, and acetylation level increased, resulting in the opening of IL‐1β gene binding chromatin structures. Glycolysis‐related transcription factors (HIF‐1α/PKM2) and other inflammatory signal transcription factors could promote IL‐1β transcription. Even when glycolytic metabolism is inhibited, the high acetylation of histone is also conducive to the transcription of IL‐1β.

When the effect of SAHA on IL‐1β expression was investigated, an unexpected phenomenon was also discovered. Macrophages were pretreated with a high concentration of SAHA for 12 hr before LPS/IFN‐γ stimulation. It was found that LPS/IFN‐γ‐mediated glycolysis metabolic conversion in macrophages was significantly inhibited after SAHA pretreatment (Fig. 7a–c). On the contrary, compared with the placebo group, SAHA‐pretreated macrophages showed more prominent oxidative phosphorylation (Fig. 7d–f). Simultaneously, WB detection showed that the expressions of glycolysis‐related protein GLUT‐1 and LDHA were significantly inhibited, and mitochondrial‐related proteins, such as COX IV and VDAC, were significantly increased (Fig. 7g). Direct mitochondrial staining with MitoTracker showed that the SAHA‐pretreated group had a large amount of mitochondrial retention after LPS/IFN‐γ stimulation (Fig. 7h). These results also indicated that SAHA‐pretreated macrophages showed more prominent oxidative phosphorylation. Although glycolytic metabolism was inhibited by SAHA, IL‐1β expression level was significantly higher than that in the placebo group (Fig. 7i). In order to eliminate the effect of the drug, TSA (a classic SAHA‐like HDAC inhibitor) was used to pretreat macrophages before LPS/IFN‐γ stimulation. Like the SAHA‐pretreatment group, the results showed that IL‐1β expression also significantly increased in the TSA pretreatment group (Fig. 7i). In summary, these results suggested that the effect of histone acetylation (H3K9 acetylation) on IL‐1β was at the downstream of glycolysis regulation, and further determined IL‐1β expression.

## Discussion

This study showed that glycolysis‐related histone acetylation in macrophages was essential for IL‐1β expression under LPS/IFN‐γ stimulation. More specifically, the levels of histone H3K9 acetylation and IL‐1β expression were observed changing parallel to the changes in glycolysis metabolism with the prolongation of duration of LPS/IFN‐γ stimulation. Subsequently, our study revealed that glycolysis was a trigger for histone H3K9 acetylation level and IL‐1β expression. Further studies showed that SAHA, an HDAC inhibitor, could reverse the IL‐1β reduction induced in the case of glycolysis inhibition by increasing histone acetylation; thus, upregulation of histone acetylation is an important mediator for glycolysis‐induced IL‐1β expression. We also observed that IL‐1β expression was significantly increased under the condition of SAHA pretreatment, even if glycolysis conversion was inhibited, suggesting the crucial role of histone acetylation in this process. Taken together, our study indicated that glycolysis‐mediated histone acetylation is a key element for upregulated IL‐1β expression in macrophages stimulated by LPS/IFN‐γ.

The metabolic conversion of macrophages under LPS stimulation played an indispensable role in the functional differentiation of macrophages.^1^, ^28^ Previously, glycolysis metabolism in M1 macrophages was assumed to be closely associated with phagocytosis and killing of pathogens by macrophages.^1^ Further studies showed that glycolysis and its related metabolic enzymes were involved in the expression regulation of some inflammatory factors. For example, Tannahill *et al.* and other scholars showed that glycolysis was indispensable for IL‐1β expression, which might be related to the enhancement of HIF‐1α stability and further promotion of IL‐1β transcription.^4^, ^11^, ^13^, ^29^ Wang *et al.* ^12^ also confirmed again that the key enzyme of glycolysis, PKM2, could enter the nucleus and form a stable complex with HIF‐1α, and then promote IL‐1β transcription as increased succinylation level. Inhibition of glycolysis could significantly decrease IL‐1β expression^11^ and, in the present study, similar results were observed. However, it was found that inhibition of glycolysis and subsequent IL‐1β expression could be reversed after SAHA intervention. Therefore, we believed that in addition to the effect on IL‐1β associated with transcription factors, glycolysis could directly affect IL‐1β transcriptional expression by changing its transcription‐associated histone structures (H3K9 acetylation).

Changes in chromatin structures could be involved in regulation of the transcriptions of some inflammatory factors.^30^, ^31^ Histone acetylation modification could activate chromatin structures and facilitate transcriptions of related genes.^25^, ^32^ In recent years, studies showed that histone modification could affect the transcription and verification levels of inflammatory cells and related inflammatory factors.^25^ For example, in rheumatoid arthritis fibroblast‐like synoviocytes, pan‐HDACi has been reported to inhibit the expression of inflammatory genes, including IL‐1β‐induced inflammatory genes.^33^ Furthermore, in RAW264.7 cells, SAHA significantly reduced the expression of TNF‐α and IL‐6 in macrophages after LPS stimulation;^34^ TSA also reduced the IL‐1β level in the serum of a mice model of post‐influenza pneumonia but, interestingly, the acetylation level of histone H3 was not affected.^35^ Additionally, in a rat model of neuroinflammation, induced by LPS, a negative correlation was reported to exist between the high acetylation level of histones and the expression of IL‐1β by exogenous long‐term acetate supplementation.^36^ However, evidence regarding the causal relationship between the acetylation level of histones and IL‐1β expression is still limited.^36^ There are also controversial views on the effects of HDACi on inflammation. For example, specific inhibition of HDAC8 was found to increase the level of H3K27 acetylation, which could reverse the inhibitory effect of anthrax lethal toxin on IL‐1β.^37^ Our present findings are in line with a previous report that the HDACs (except HDAC1) transcription is temporarily inhibited and negatively correlated with the transcriptional expression of pro‐inflammatory genes after LPS stimulation in macrophages,^34^ and H3K9 hyperacetylation could promote IL‐1β transcription.^18^

Changes in chromatin structures were also closely associated with metabolic types.^6^ Lactic acid, a product in the glycolysis process, could act as a weak HDAC inhibitor, and might act as a transcriptional regulator in influencing gene transcriptions by modifying chromatin structures.^19^ Therefore, we hypothesized and proved that glycolysis not only affected IL‐1β‐associated transcription factors, such as HIF‐1α and PKM2, but also regulated IL‐1β‐associated transcription factors by affecting histone acetylation through regulating HDACs and acetyl‐CoA donors. In our study, we first observed that 2DG reversed LPS/IFN‐γ‐mediated transcriptional inhibition of HDACs, accompanied by a decrease in the expression of IL‐1β. In addition, we also detected acetyl‐CoA donors (such as acetate and citrate), and found that the glycolysis conversion process of macrophages was accompanied by a significant increase in acetate and citrate contents. Interestingly, citrate was significantly reduced by 2DG intervention, in contrast acetate was not significantly affected, suggesting that glucose may not be the major source of acetate during the macrophage glycolysis conversion process. These above findings allowed us to conclude that HDACs transcription and acetyl‐CoA donors are closely associated with the glycolysis metabolism, which eventually regulates the expression of IL‐1β by changing histone acetylation. Modifications of other histone loci/other histone modification types and macrophage function/phenotype with the current experimental setting were not involved in this study.

Stammler *et al.* ^38^ described that the treatment with HDACi (SAHA 50 mg/kg body weight) in a dextran sodium sulphate‐induced colitis mice model resulted in a strong increase in intestinal IL‐1β. In this experiment, we coincidentally found that SAHA resulted in different end effects on IL‐1β mRNA expression under different intervention modes (pre‐ or simultaneous stimulation with LPS/IFN‐γ) in macrophages. Based on multiple previous reports on the association between glycolysis and IL‐1β mRNA expression,^1^, ^11^, ^12^, ^39^ we tested the hypothesis that HDACi could ultimately inhibit the expression of IL‐1β by blocking the glycolysis metabolic conversion. Unexpectedly, we found that even if macrophage glycolysis conversion was significantly inhibited by pretreatment with SAHA under LPS/IFN‐γ stimulation, IL‐1β mRNA was still significantly increased, opposite to the results of 2DG, a classic glycolysis inhibitor, which induced the inhibition of IL‐1β expression. One potential interpretation on the divergent effects of SAHA and 2DG might be their divergent impact on histone acetylation independent of their effects on glycolysis metabolism, 2DG reduced H3K9 acetylation while SAHA increased H3K9 acetylation, which might be responsible for the observed upregulation of IL‐1β mRNA expression post‐SAHA. This also suggested that glycolytic metabolism mode in M1 macrophages not only stabilized and promoted the transcription of glycolysis‐associated transcription factors of IL‐1β, but also, more importantly, maintained IL‐1β‐associated histone hyperacetylation, which might be a precondition for IL‐1β transcription (Fig. 7j). In conclusion, we found that glycolysis‐mediated histone acetylation is a key element for upregulated IL‐1β expression in macrophages stimulated by LPS/IFN‐γ.

## Disclosures

The authors declare no competing interests.

## Supporting information


**Figure S1.** Expression of mitochondrial RNA encoded by mitochondrial DNA were assessed by RT‐PCR in BMDMs activated by LPS/IFN‐γ.
**Figure S2.** Dose dependent effects of LPS and IFN‐γ on glycolysis metabolic conversion and the acetylation level of H3K9 in macrophages.
**Figure S3.** Changes of cellular pyruvate and lactate content during glycolytic transformation in LPS/IFN‐stimulated macrophages.
**Figure S4.** Augment of acetylation level of H3K9 and IL‐1β expression in Sirt5 KO macrophages activated by LPS/IFN‐γ and effects of LPS and IFN‐γ combined with or without SAHA on IL‐1β, TNF‐α, IL‐6 and NOS2 expression.
