# Supplementary material for: Histone hyperacetylation mediates enhanced IL‐1β production in LPS/IFN‐γ‐stimulated macrophages
Source: Immunology. 2020 Apr 7;160(2):183–97. doi: 10.1111/imm.13183 (PMC7218666; doi:10.1111/imm.13183)
Supplement: Supplementary file 1 — Figure S1. Expression of mitochondrial RNA encoded by mitochondrial DNA were assessed by RT‐PCR in BMDMs activated by LPS/IFN‐γ. Figure S2. Dose dependent effects of LPS and IFN‐γ on glycolysis metabolic conversion and the acetylation level of H3K9 in macrophages. Figure S3. Changes of cellular pyruvate and lactate content during glycolytic transformation in LPS/IFN‐stimulated macrophages. Figure S4. Augment of acetylation level of H3K9 and IL‐1β expression in Sirt5 KO macrophages activated by LPS/IFN‐γ and effects of LPS and IFN‐γ combined with or without SAHA on IL‐1β, TNF‐α, IL‐6 and NOS2 expression. [file IMM-160-183-s001.pdf]

Figure S1

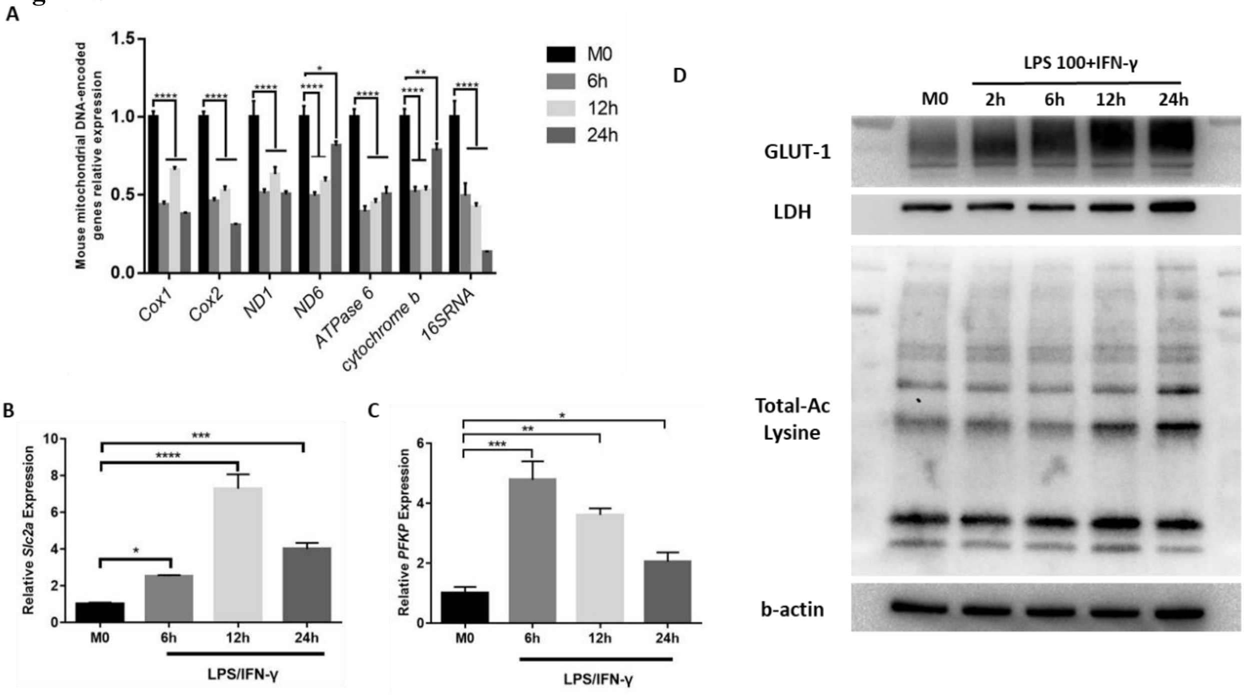

Figure S1 A: Expression of mitochondrial RNA encoded by mitochondrial DNA were assessed by RT-PCR in BMDMs activated by LPS/IFN-γ. ND1, NADH dehydrogenase subunit 1; ND6, NADH dehydrogenase subunit 6; cytB, cytochrome b; cox1, Cyt c oxidase subunit 1; cox2, Cyt c oxidase subunit 2; ATP6, ATP synthase F0 subunit 6; 16S RNA. $*P < 0.05$, $**P < 0.01$, $****P < 0.0001$. All samples were analyzed as fold change against M0 inactivated control samples. n = 3 independent measurements. Data are presented as mean±SD. **B and C:** mRNA levels of Slc2a, and Pfkp3 were assessed by RT-PCR in BMDMs activated by LPS/IFN-γ. $*P < 0.05$, $**P < 0.01$, $***P < 0.001$, $****P < 0.0001$. All samples were analyzed as fold change against M0 inactivated control samples. n = 3 independent measurements. Data are presented as mean±SD. **D:** Representative WB of GLUT-1, LDHA, and total protein acetylated lysine (Total-Ac Lysine) in BMDMs induced by LPS/IFN-γ. n = 3 independent measurements.

Figure S2

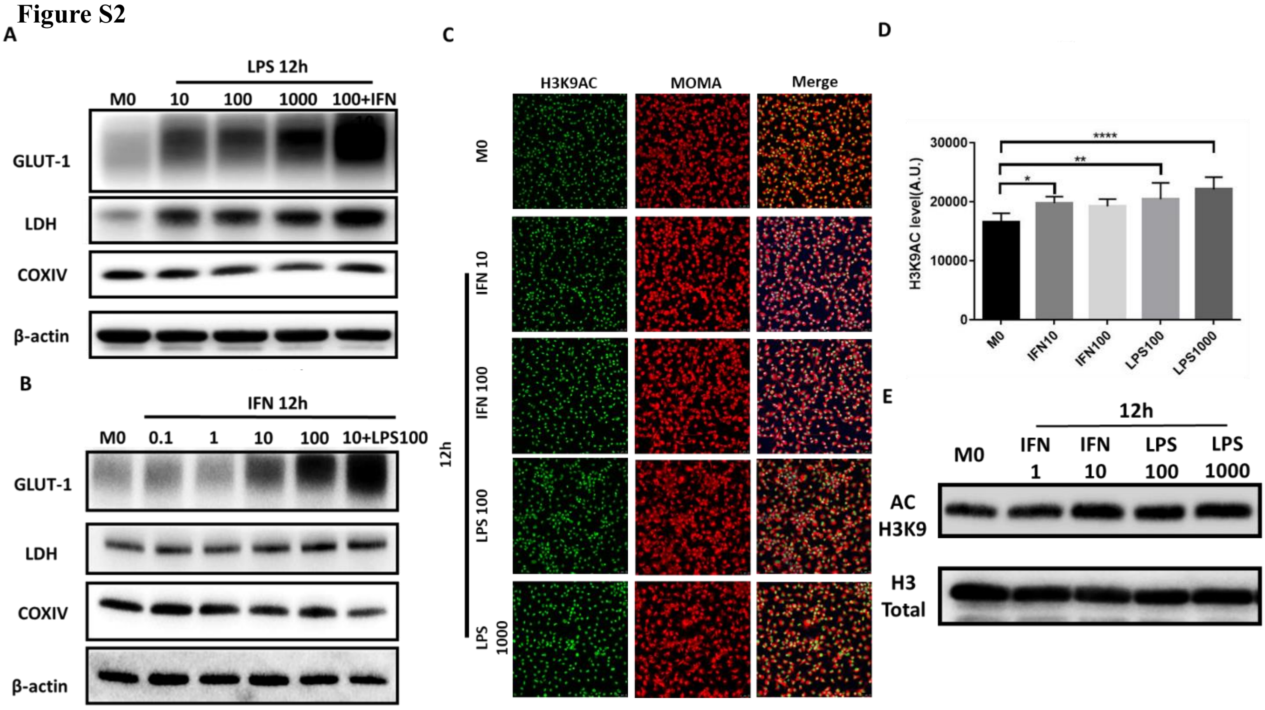

Figure S2 Dose dependent effects of LPS and IFN-γ on glycolysis metabolic conversion and the acetylation level of H3K9 in macrophages. A and B: Representative WB of GLUT-1, LDHA and COXIV levels in BMDMs 12 h activated by LPS(10,100,1000 and 100+INF-γ10 ng/mL) or IFN-γ(0.1,1,10,100 and 10+LPS100 ng/mL). n = 3 independent measurements. **C:** Representative immunofluorescence images of AC-H3K9 during differentiation of BMDMs activated by LPS (100 and 1000 ng/mL) or IFN-γ (10 and 100 ng/mL). Green, AC-H3K9; red, MOMA2 and blue, DAPI. A.U. indicates arbitrary units. Scale bar, 25um.**D:** Quantification of AC-H3K9 immunostaining in BMDMs as in (C) at induced by LPS (100 and 1000 ng/mL) or IFN-γ (10 and 100 ng/mL). *P < 0.05, **P < 0.01, **** P < 0.0001. n = 3 independent measurements. Data are presented as mean±SD. **E:** Representative WB of AC-H3K9 in BMDMs at induced by LPS (100 and 1000 ng/mL) or IFN-γ (1 and 10 ng/mL). n = 3 independent measurements.

Figure S3

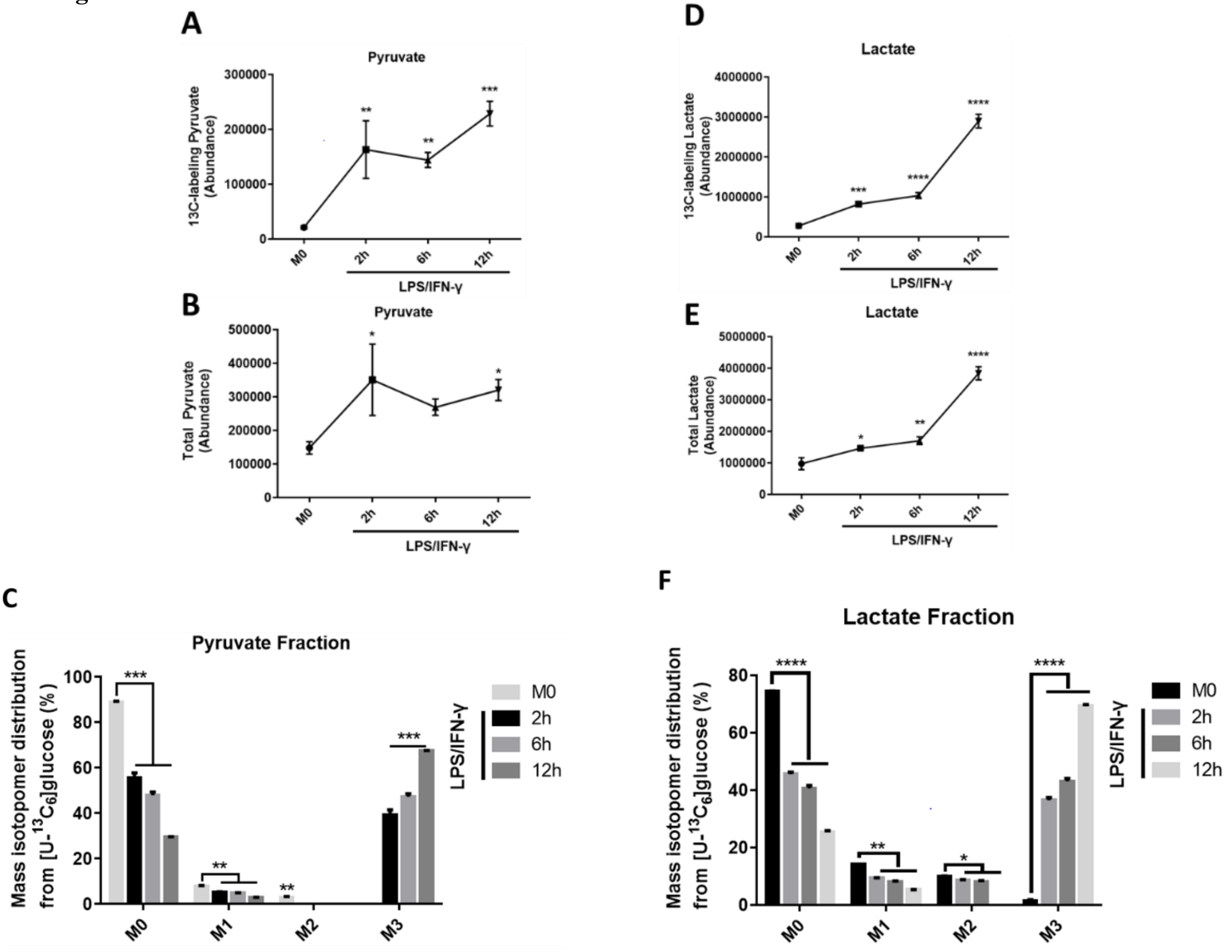

Figure S3 Changes of cellular pyruvate and lactate content during glycolytic transformation in LPS/IFN-stimulated macrophages. A and B: Cellular 13C-labeling and total pyruvate content examined by GC-MS in LPS/IFN-stimulated macrophages. **C:** Mass isotopologue analysis of pyruvate in LPS/IFN-stimulated macrophages cultured with [U-13C6]-glucose. M (0-3): the number of labeled carbons; *:P < 0.05, **P < 0.01, ***P < 0.001; n = 3 cultures from a representative experiment; **D and E:** Cellular 13C-labeling and total lactate content examined by GC-MS in LPS/IFN-stimulated macrophages; **F:** Mass isotopologue analysis of lactate in LPS/IFN-stimulated macrophages cultured with [U-13C6]-glucose. M (0-3): the number of labeled carbons; *P < 0.05, **P < 0.01, ***P < 0.001, **** P < 0.0001; n = 3 cultures from a representative experiment; Data are presented as mean±SD.

Figure S4

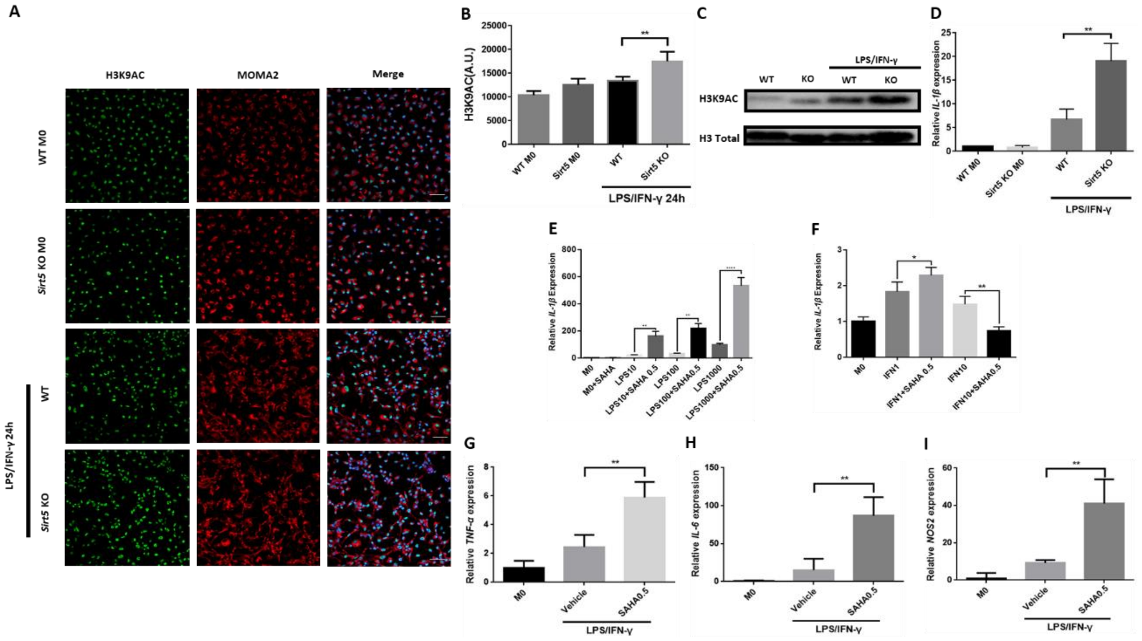

Figure S4 Augment of acetylation level of H3K9 and IL-1β expression in *Sirt5* KO macrophages activated by LPS/IFN-γ and effects of LPS and IFN-γ combined with or without SAHA on IL-1β, TNF-α, IL-6 and NOS2 expression.A: Representative immunofluorescence images of histone acetylation at AC-H3K9 during differentiation of BMDMs isolated from WT or *Sirt5* KO mice activated by LPS/IFN-γ 24 h. Green, AC-H3K9; red, MOMA2 and blue, DAPI. A.U. indicates arbitrary units. Scale bar, 50 um.**B:** Quantification of AC-H3K9 immunostaining in WT or *Sir*t5 KO BMDMs as in (A) activated by LPS/IFN-γ 24 h. **P < 0.01. n = 3 independent measurements. Data are presented as mean±SD. **C:** Representative WB of AC-H3K9 in WT or *Sirt5* KO BMDMs induced by LPS/IFN-γ 24 h. n = 3 independent measurements. **D:** mRNA levels of *IL-1β* were assessed by RT-PCR in WT or *Sirt5* KO BMDMs activated by LPS/IFN-γ 24 h. **P < 0.01. All samples were analyzed as fold change against WT M0 inactivated control samples. n = 3 independent measurements. Data are presented as mean±SD. **E and F:** mRNA levels of *IL-1β* were assessed by RT-PCR in BMDMs activated by LPS (10,100, and 1000 ng/mL) or IFN-γ (1 and 10 ng/mL) with or without SAHA (0.5uM) 12 h. n = 3 independent measurements. *P < 0.05, **P < 0.01. All samples were analyzed as fold change against M0 inactivated control samples. Data are presented as mean ±SD. **G, H and I:** mRNA levels of *TNF-α, IL-6* and *NOS2* were assessed by RT-PCR in BMDMs activated by LPS/IFN-γ with or without SAHA (0.5uM) 12 h. n = 3 independent measurements. **P < 0.01. All samples were analyzed as fold change against M0 inactivated control samples. Data are presented as mean ±SD.
